# Use of Temperature, Humidity, and Slaughter Condemnation Data to Predict Increases in Transport Losses in Three Classes of Swine and Resulting Foregone Revenue

**DOI:** 10.3389/fvets.2017.00067

**Published:** 2017-05-11

**Authors:** Erik Peterson, Marta Remmenga, Amy D. Hagerman, Judy E. Akkina

**Affiliations:** ^1^United States Department of Agriculture, Animal and Plant Health Inspection Service, Veterinary Services, Fort Collins, CO, USA

**Keywords:** swine surveillance, swine condemnations, in-transit swine death, cold stress, heat stress, cull sow, roaster swine, market swine

## Abstract

The United States Department of Agriculture (USDA) Animal and Plant Health Inspection Service (APHIS) conducts weekly surveillance of slaughter condemnation rates to provide early warning for emerging diseases and to monitor health trends in swine. Swine deaths in-transit are an animal welfare concern and represent lost revenue for the swine industry. This retrospective observational study used ambient temperature and humidity data from weather stations near United States slaughter plants collected from 2010 to 2015 to predict the incidence and risk of death among swine in-transit and just prior to slaughter. The risk of death for market swine at a heat index (HI), which combines the effects of temperature and humidity, indicating moderately hot weather conditions between 85 and 92°F was 1.37 times greater than that of the baseline temperature range of 54–79°F. The risk of death for cull sows at an HI between 85 and 92°F was 1.93 times greater than that of average temperatures ranging from 54 to 79°F. Roaster swine (weigh < 220 lbs and often used for whole carcass roasting), however, had 0.80 times the risk when the HI was 85–92°F compared to a baseline temperature of 54–79°F. The risk of death for roaster swine at a minimum temperature between 40 and 50°F was 1.21 times greater than that of average temperatures ranging from 54 to 79°F. The risk of death for market swine at a minimum temperature range of 40–50°F was 0.97 times that of average temperatures ranging from 54 to 79°F. And for cull sows, the risk of death at a minimum temperature range of 40–50°F was 0.81 times the risk at the average temperature ranging from 54 to 79°F. Across the study period, cumulative foregone revenue, or revenue not realized due to swine condemnations, for all swine was $18.6 million and $4.3 million for cold temperatures and high HI ranges above the baseline, respectively. Marginal foregone revenue per hog in hotter months is higher due to seasonal peaks in swine prices. As a result of this study, the USDA-APHIS swine condemnation surveillance can incorporate weekly estimated HI values and ambient temperature data for slaughter establishments to provide additional information for analysts investigating signals (noteworthy increases above baseline) for “dead” condemnations. This study suggests that current mitigation measures are often not sufficient to prevent swine deaths due to ambient temperature extremes.

## Introduction

Pig deaths in-transit or in lairage pens just prior to slaughter (“dead” condemnations) are an animal welfare issue, a potential indicator of disease, and an economic loss to the producer, the transporter, and slaughter establishments (1). Factors associated with market swine death during transport or at the slaughter establishment prior to slaughter include genetics, handling before they are transported, disease, poor health, stress, and conditions during transport (2). High ambient temperature combined with high humidity is one of the most important environmental conditions contributing to in-transit death loss of market swine destined for slaughter (1). Pigs are prone to heat stress because they have a limited ability to dissipate body heat since they do not sweat and their high sub-cutaneous fat inhibits heat transference to the environment. Low temperatures can also lead to increases of in-transit death loss of market swine (3). Other conditions during transport that affect the mortality and morbidity of market pigs include length of the journey, density of pigs on the trailer, the type of trailer, the amount and condition of bedding, and wait time at the slaughter establishment (2, 4, 5).

As part of an early warning surveillance system, the United States Department of Agriculture (USDA) Animal and Plant Health Inspection Service (APHIS) conducts weekly surveillance of condemnation rates for specific reasons at federally inspected swine slaughter establishments across the country. The purposes of this surveillance are to identify noteworthy increases (signals) above baseline condemnation rates/counts in near real-time that may indicate the emergence of disease and to monitor health trends. These signals may warrant further investigation to determine their cause. Surveillance analysts have noted that signals for the condemnation reason “dead” are most frequent during the summer months.

Historically in the United States (US), the incidence of dead pigs at USDA-inspected slaughter plants was reported to be 0.22% from 2002 to 2006 (6). Relative to market swine, there is minimal information in the literature on the effects of ambient temperature on transit deaths for roaster swine (weigh < 220 lbs and often used for whole carcass roasting) or cull sows. A better understanding of the impact of ambient temperature on swine condemnations for the reason “dead” can improve the interpretation of signals that occur during analysis of surveillance data and can help guide the need for follow-up investigations associated with these signals. Further, understanding the consequential foregone revenue for producers may incentivize temperature stress mitigation strategies and improved swine welfare in transit during times of extreme temperatures.

The purpose of this study was to explore the effects of temperature and humidity on swine deaths in-transit and at the slaughter facility just prior to slaughter, as well as the associated foregone revenue from temperature-related swine deaths. We hypothesized that extreme temperatures would increase the “dead” condemnation rate (CR) in all swine classes. The effects of high ambient temperature and humidity were examined using the heat index (HI)—a measure of the combined effects of temperature and humidity of the air, also known as the apparent temperature—and minimum ambient temperature near the slaughter establishment during the week of slaughter according to swine class (market, roaster, and cull sows).

## Materials and Methods

The USDA’s Food Safety and Inspection Service (FSIS) Public Health Information System (PHIS) contains information about swine inspection at FSIS-inspected slaughter establishments. Variables in the database used for this study include the week of condemnation or slaughter, swine class, reason for condemnation, number of head condemned by week, and number of head processed by week. APHIS has a Memorandum of Understanding with FSIS to allow access to this data in PHIS. Due to the constraints of our software tools capabilities, we limited our analysis to 6 years of PHIS slaughter and condemnation data from January 2010 to December 2015.

In the database, there was no information on evaluation of the pigs during loading, type of truck, and distance or duration of transport to the slaughter plant. In North America (NA), multiple types of trucks are used to transport swine and in general a large percentage of slaughter pigs are delivered in large semi-trucks, either straight double deck or potbellies (7). Operations with grower/finisher sites participating in the USDA National Animal Health Monitoring System Swine 2012 study reported that the closest average distance traveled to slaughter for market pigs was 22.8 miles and the farthest average distance was 79.5 miles (8). Therefore, it is likely that the majority of market pigs in our study traveled for less than 4 h (7). Information about the distance traveled to slaughter for roaster pigs or cull sows in our study is not available. A small percentage of pigs in the US may be transported long distances and may spend more than 24 h in transit. Under US federal, law animals must be humanely offloaded after 28 h in transit and can then eat, drink, and rest for at least 5 h (9). Regarding the evaluation of pigs loaded, in the US accredited veterinarians must ensure that swine moving in interstate commerce meet all State and Federal animal health requirements. Accredited veterinarians are those that are licensed in the State where they practice and have received additional accreditation training and certification by APHIS to perform certain activities, such as issuing a Certificate of Veterinary Inspection (CVI). Accredited veterinarians are expected to use their professional judgment based on their veterinary training and experience to determine if any abnormality in physical condition or bodily function is suggestive of clinical signs of communicable disease before issuing a CVI (10). Swine moving to slaughter in the State where they were raised would typically not need a CVI. Pigs that will not pass ante-mortem or post-mortem inspection will be condemned and result in a financial loss; therefore, producers are less likely to include such animals in a load.

The effects of the HI and minimum temperature on three classes of swine were studied. HI was chosen after running regression models and finding that humidity explained a significant amount of the variation in addition to temperature. Various temperature humidity indices have been used in the literature (11); however, not all weather stations reported the needed variables to calculate these, therefore we used the HI.

Roaster swine are small, non-conforming to standard market swine specifications (weigh < 220 lbs) and are often used for whole carcass roasting. Market swine typically weigh between 220 and 260 lbs. Cull sows are the female breeding swine removed from the farrowing herd, with an average weight above 400 lbs. We did not include cull boars in our study because their numbers in the data were too low for meaningful analysis.

**Figure 1:**
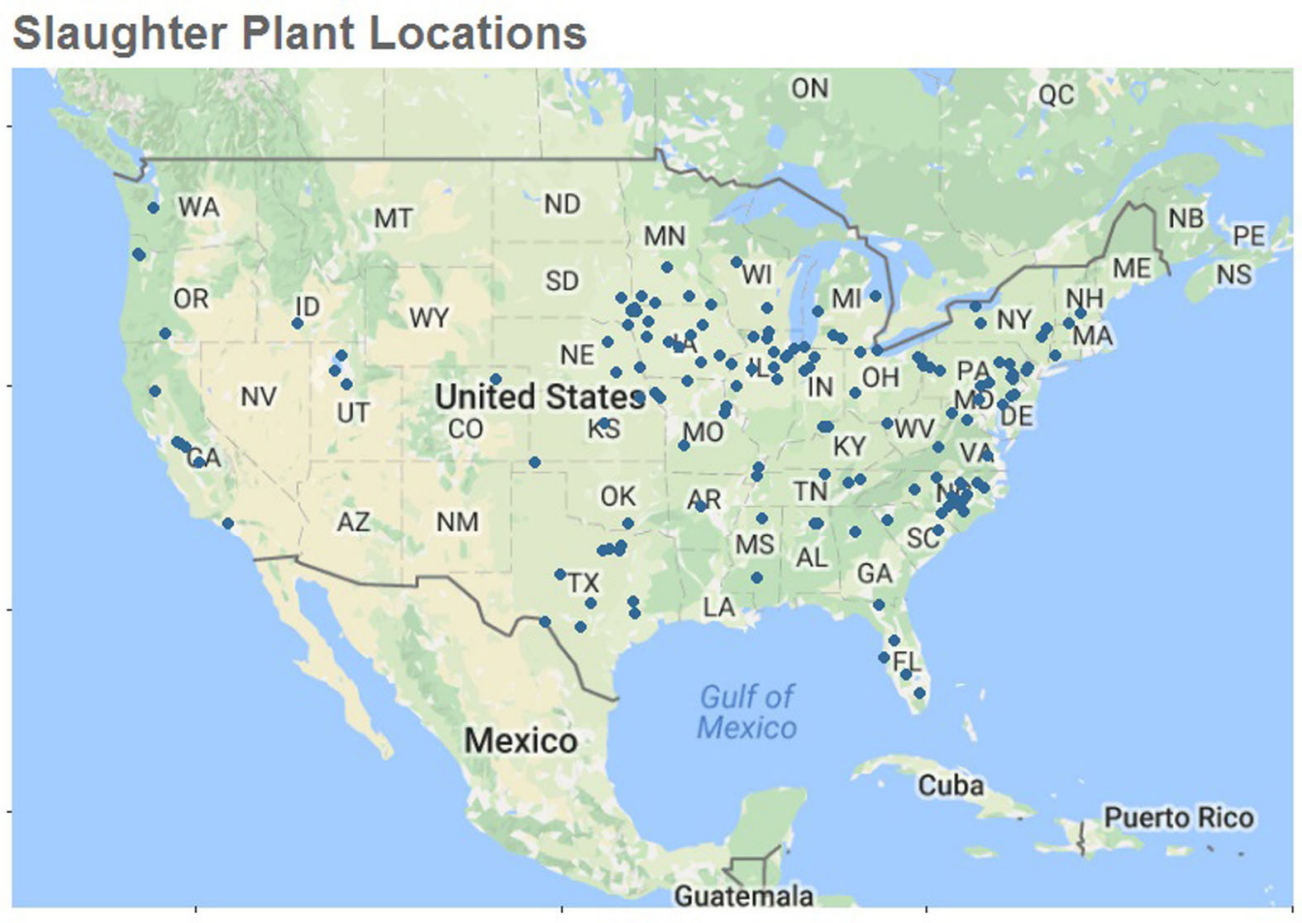
**Location of the 149 slaughter plants used to examine temperature, humidity, and slaughter condemnation data to predict increases in transport losses in United States Swine in 2010–2015**. Only slaughter plants with temperature and humidity data within 100 miles of plant location were used for this study.

After merging tables and assigning weather data, 8% of slaughtered swine were lost from our original dataset. Remaining was a dataset that included 4.2 million roaster swine, 619.2 million market swine, and 17.4 million cull sows from 149 different federally inspected slaughter establishments throughout the US during our study period. Using R package ggmap (12), Figure 1 was created to show the slaughter plants used in our study. The majority of our plants are in the eastern part of the country. About 50% of the swine in our dataset came from the largest 9 plants, the remaining 140 plants produced the rest.

“Dead” loss ratios (DLR) were calculated for all swine each month by dividing the number of “dead” condemnations by the total swine and multiplying by 100. This provided the incidence of “dead” condemnations over the study period.

**Figure 2:**
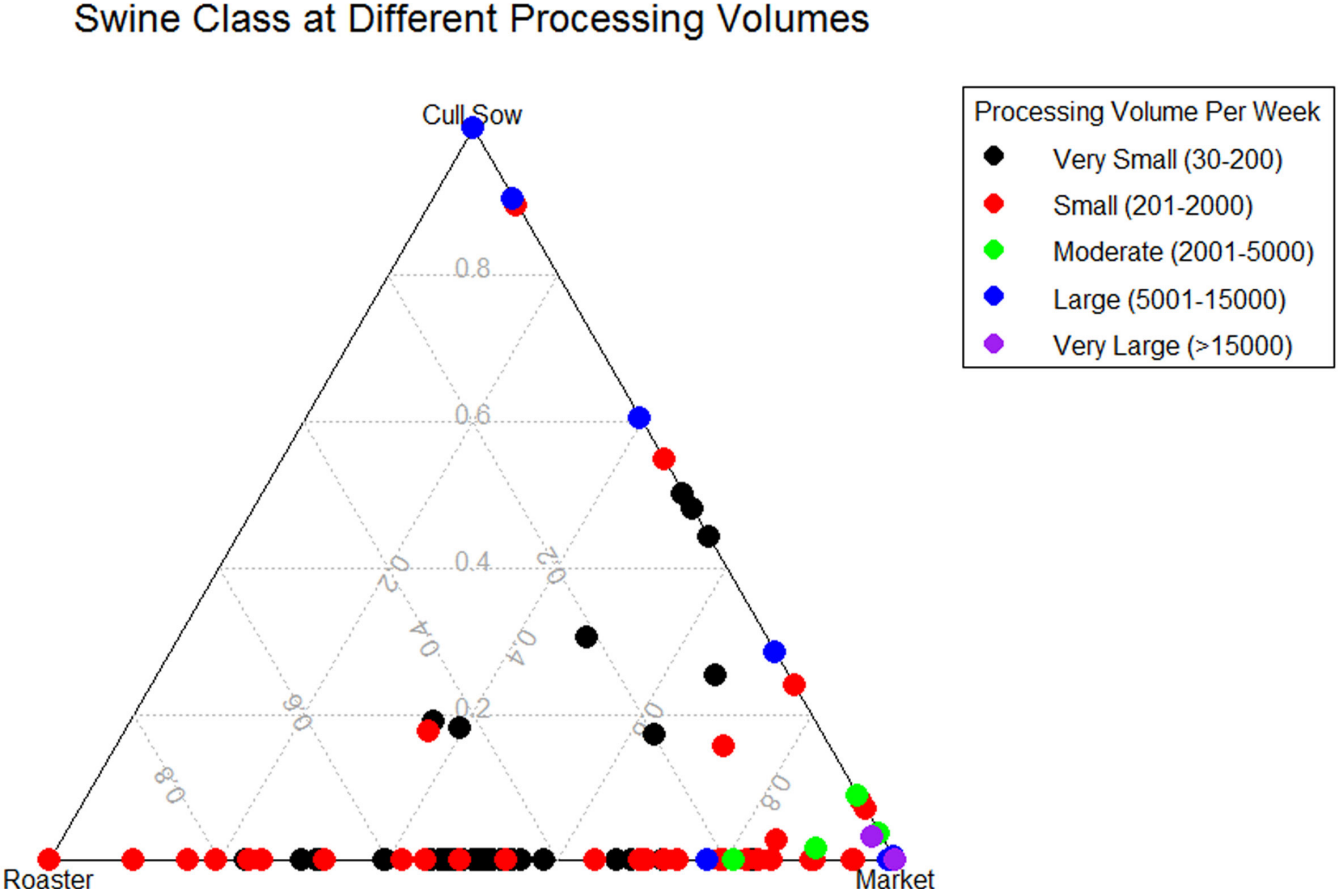
**Ternary plot showing the slaughter proportion of three different swine classes (Roaster, Market, Cull Sow) by five plant processing volume per week categories in 149 slaughter plants with temperature and humidity data within 100 miles of plant location for United States Swine from 2010 to 2015**.

Establishment processing volume (number of head slaughtered) was recorded for each week and used as a predictor variable for condemnations to fit a cubic smoothing spline. Observing this spline, we were able to break up this continuous variable into five categories to group values with similar effects on condemnations. This new categorical variable was used as a random effect in our model to help account for the variability among the different plant sizes. A ternary plot showing the proportions of each swine class slaughtered for all 149 slaughter plants included in our study can be seen in Figure 2. The largest processing volume plants dealt almost exclusively with market swine, other large processors had a mixture of cull sows and market. Those plants with the lowest volume generally processed all three swine classes, but roaster swine came almost entirely from these. In Table 1, the number of weeks, the average slaughtered each week, and the total amount slaughtered in our period of study is shown for each processing volume category.

**Table 1:** **The number of observations (weeks), mean slaughter count, and total swine slaughtered for each processing volume category in 149 slaughter plants with temperature data within 100 miles of plant location for United States Swine 2010–2015**.

Processing volume category (swine per week)	Number of observations (weeks)	Mean slaughter count	Total swine slaughtered
VS (30–200)	9,564	101	975,284
S (201–2,000)	16,403	719	11,790,821
M (2,001–5,000)	4,485	2,565	11,502,422
L (5,001–15,000)	4,272	6,971	29,780,745
VL (>15,000)	8,931	65,717	586,921,865

Weather information was taken from the website Wunderground.com via the R package “weatherData” (13). Using the Haversine formula, which calculates the distance on a sphere using longitude and latitude, we recorded the closest weather stations for each slaughter establishment. To estimate the temperature and humidity for each slaughter establishment for each week of slaughter data, the average daily temperature, minimum daily temperature, and humidity were taken from the closest 1–3 weather stations within 100 miles.

The HI was calculated using the average temperature and humidity for the week. HI is expressed as the apparent temperature in degrees Fahrenheit (°F) at temperatures above 80°F. “The computation of the HI is a refinement of a result obtained by multiple regression analysis carried out by Lans P. Rothfusz and described in a 1990 National Weather Service Technical Attachment (SR 90-23).”^1^

Once the HI values were obtained, they were categorized similarly to processing volume. We fit a cubic smoothing spline to the data, but this time included an offset term equaling the log of total swine (all swine brought to the establishment for that week). This effectively weighted our observations based on volume so that slaughter plants that processed more swine were more influential in our regression. We called the three temperature categories for HI moderate, hot, and very hot, which included temperatures within the ranges 80–84°F, 85–92°F, and 93–106°F, respectively.

To study the effect of cold temperatures on swine, all records with an average weekly minimum temperature of 50 or below were categorized the same way as the HI values. A cubic spline was fit to the data for each swine class with an offset term of log of total swine. We called the three temperature categories for minimum temperature very cold, cold, and cool, which included temperatures within the ranges −17 to 9°F, 10–39°F, and 40–50°F, respectively.

The baseline temperatures ranged from a minimum of 51 to an average of 79°F. This range was chosen because it excluded all of the records from our HI and minimum temperature datasets. Among weeks where the average minimum temperature was above 50°F, the lowest average temperature was 54°F. So our baseline category was set to average temperatures of 54 to 79°F. To determine the estimated losses in each temperature category within the minimum temperature and HI groups, a zero-inflated negative binomial generalized linear mixed model (ZINBGLMM) was used. This model was chosen because roughly half of our observations had no condemnations and there was over-dispersion. These observations with no condemnations came primarily from slaughter establishments with fewer than 1,000 head processed per week. In this model, the number of pigs that were condemned for the reason “dead” per week was used as a response variable with our categorical predictor variables, swine class and temperature category or HI. Because plants that processed different amounts of swine each week had varying facility qualities and procedures, we set volume processed as our random effect to help account for that variability. This was done using R with the “glmmADMB” package (14), which was chosen due to its ability to include both an offset term and a random effect term.

The attributable risk (AR) for each temperature category and swine class was calculated by taking the difference in estimated condemnation risk of the baseline and the estimated condemnation risk of the exposed groups. Doing this gives us the change in risk of “dead” condemnation when moving from our baseline to our various temperature categories.

Once the estimated losses due to each temperature category were determined, we estimated the foregone revenue associated with high or low temperatures. Data from weeks with a minimum temperature above 50 and an average temperature below 80 were used as a comparison to estimate the additional losses associated with extreme temperatures. Another ZINBGLMM was created to predict the amount condemned due to reasons other than temperature. This type of model was chosen for the same reasons as previously mentioned, but used all the records not included in our hot or cold models. Here, the number of pigs that were condemned for the reason “dead” per week was used as a response variable with our swine class as our predictor value. Once again, the log of total swine was used as an offset and volume processed category as our random effect. We used the resulting model to predict the amount of “dead” condemnations in our data lying outside of the baseline temperature range and compared it to the actual amount condemned. The resulting residuals were assumed to be primarily due to the increased or decreased temperature, and the sums of additional condemnations were calculated for each temperature category.

The Agriculture Marketing Service (AMS) releases national daily direct swine reports for market and breeding swine in the US. These reports were compiled by the Livestock Marketing Information Center^2^ to include the monthly average price per carcass for market and cull breeding swine types across a number of years. Roaster swine prices were not included in these reports. To estimate roaster swine prices, the monthly average dressed price per hundredweight in market swine were used instead.

Using this information, we estimated the amount of foregone revenue for market and cull sow swine due to estimated temperature-related “dead” condemnations for each slaughter plant by week. This was done by multiplying the increased number condemned as determined by our previous calculations and the average price per swine for that particular month. The foregone cost of roaster swine was calculated by taking the median roaster carcass weight (70 lbs) and dividing by the price per hundredweight for market swine, then multiplying by the increased roaster condemnations.

For categories of weekly HI, a CR was calculated for each swine class in a slaughter establishment. Multiplying the CR by 10,000, we calculated an expected incidence rate. Using the results of a ZINBGLMM, a risk ratio (RR) was calculated to determine the increased risk when comparing categories of HI. This was done by taking the predicted CR, and dividing by the reference category (54–79°F).

## Results

**Figure 3:**
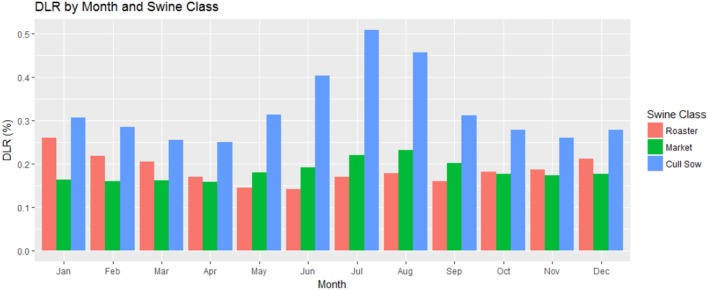
**“Dead” loss ratios by swine class and month in 149 slaughter plants with temperature and humidity data within 100 miles of plant location for United States Swine in 2010–2015**. “Dead” loss ratio calculated by number of “dead” condemnations divided by total swine multiplied by 100.

With the HI formula used in this study only valid above 80°F and our minimum temperature categories starting at 50°F, we first looked at temperature averages with our full dataset. The DLR for all swine was 0.19% (19 pigs per 10,000 slaughtered). Disaggregating by months, we find the highest DLR to be in the months with the warmest average temperature, July and August (Table 2). Disaggregating again by swine class, we find cull sows to have the highest DLR, and also that for roaster swine the DLR is highest in the winter months (Figure 3).

**Table 2:** **“Dead” loss ratios (DLR), average temperature, and temperature range by month for all swine classes in 149 slaughter plants with temperature and humidity data within 100 miles of plant location for United States Swine in 2010–2015**.

Month	DLR^a^ (%)	Avg. temp (°F)	Range (°F)
January	0.168	33.4	−7 to 78
February	0.164	35.9	0–79
March	0.163	46.9	8–81
April	0.161	56.7	27–96
May	0.183	65.4	36–96
June	0.197	72.9	48–93
July	0.228	76.9	52–96
August	0.236	74.5	51–93
September	0.204	67.5	41–92
October	0.180	56.9	30–95
November	0.176	45.8	12–88
December	0.180	38.5	−2 to 79

*^a^DLR calculated by number of “dead” condemnations divided by total swine multiplied by 100*.

Our ZINBGLMM model using HI as a predictor included 348,000 roaster swine, 24.8 million market swine, and 1.2 million cull sows at 131 different federally inspected slaughter establishments. These were the records we could apply the HI formula to. There was a significant effect (*p* < 0.05) of each HI category on the CR in all three swine classes in our model (Table 3).

**Table 3:** **Weekly condemnation rate (CR), attributable risk (AR), and expected incidence rate (EIR) of “dead” condemnations by heat index (HI) category in 149 slaughter plants with temperature and humidity data within 100 miles of plant location for United States Swine 2010–2015**.

HI^b^ (°F)	Est. prob. condemned (CR)	AR	EIR (per 10,000)
**Roaster**			
Baseline	0.00123^a^		12
Moderate	0.00173^a^	0.0005	17
Hot	0.00098^a^	−0.00025	10
Very hot	0.00015^a^	−0.00108	2
**Market**			
Baseline	0.0030^a^		30
Moderate	0.0038^a^	0.0008	38
Hot	0.0041^a^	0.0011	41
Very hot	0.0035^a^	0.0005	35
**Cull sows**			
Baseline	0.0027^a^		27
Moderate	0.0056^a^	0.0029	56
Hot	0.0052^a^	0.0025	52
Very hot	0.0061^a^	0.0034	61

*Estimated CR are based on a zero-inflated negative binomial generalized linear mixed model*.

*^a^Significant at alpha = 0.05. All HI categories are nested within swine classes. For example, a significant proportion of variability in “dead condemnations” was accounted for by the BASELINE HI category nested within the ROASTER class after accounting for the offset term, log of total swine, and all other factors in the model*.

*^b^HI categories of baseline, moderate, hot, and very hot ranges are 54–79°F, 80–84°F, 85–92°F, and 93–106°F, respectively*.

Market and cull sows were negatively impacted by an increase in HI. For every 10,000 market swine that go to slaughter in a week, our model suggests that we can expect to lose approximately 38, 41, and 35 market swine due to “dead” condemnations at HI ranges that are moderate (80–84°F), hot (85–92°F), and very hot (93–106°F), respectively. For every 10,000 cull sows that go to slaughter, we can expect to lose approximately 56, 52, and 61 at moderate, hot, and very hot HI ranges, respectively. The AR for the hottest HI category for market swine was 0.0005, and for cull sows it was 0.0034. So moving the market and cull sows from the hottest temperatures into our baseline temperature range would reduce the “dead” CR.

However, in roaster swine, there was a decrease in condemnations at hot and very hot HI ranges compared to baseline. Our model predicts there will be approximately 17, 10, and 2 “dead” condemnations for every 10,000 roaster swine that go to slaughter in a week at moderate, hot, and very hot HI ranges, respectively. The AR for the hottest HI category for these swine was −0.00108, meaning there is actually a decrease in condemnation risk when moving from our baseline to the hottest temperature category.

Setting our baseline to be the observations with average temperatures ranging from 54 to 79, the risk of the moderate HI category for roaster swine is 1.41 times greater than the baseline category (Table 4). The hot and very hot HI weekly averages have a risk of 0.80 and 0.12 times that of the baseline, respectively.

**Table 4:** **The risk ratio (RR) and 95% confidence interval range of “dead” condemnations by heat index (HI) category in 149 slaughter plants with temperature data within 100 miles of plant location for United States Swine 2010–2015**.

HI (°F)^a^	RR	Confidence interval
**Roaster**		
Baseline	1.00	
Moderate	1.41	(1.27, 1.56)
Hot	0.80	(0.72, 0.89)
Very hot	0.12	(0.10, 0.14)
**Market**		
Baseline	1.00	
Moderate	1.27	(1.13, 1.42)
Hot	1.37	(1.22, 1.54)
Very hot	1.17	(1.03, 1.33)
**Cull sows**		
Baseline	1.00	
Moderate	2.07	(1.86, 2.31)
Hot	1.93	(1.73, 2.15)
Very hot	2.26	(2.00, 2.55)

*^a^HI categories of baseline, moderate, hot, and very hot ranges are 54–79°F, 80–84°F, 85–92°F, and 93–106°F, respectively*.

For market swine with an average HI within moderate, hot, and very hot range, the risk of condemnation due to “dead” is 1.27, 1.37, and 1.17 times greater, respectively. For breeding swine with an average HI in the moderate, hot, and very hot range, the risk of condemnation due to “dead” is 2.07, 1.93, and 2.26 times greater than that of our baseline, respectively.

Our ZINBGLMM model using minimum temperature as a predictor included 2.3 million roaster swine, 386.1 million market swine, and 10.7 million cull sows at 145 different federally inspected slaughter establishments. There was a significant effect (*p* < 0.05) of minimum temperature on the CR in each of the swine classes in our model, with roaster swine in the 10–39°F (cold) category being the exception (*p* = 0.073) (Table 5).

**Table 5:** **Weekly condemnation rate (CR), attributable risk (AR), and expected incidence rate (EIR) of “dead” condemnations by temperature index category in 149 slaughter plants with temperature data within 100 miles of plant location for United States Swine 2010–2015**.

Min temp^b^ (°F)	Est. prob. condemned CR	AR	EIR (per 10,000)
**Roaster**			
Baseline	0.00123^a^		12
Cool	0.00149^a^	0.00026	14
Cold	0.00191	0.00068	19
Very cold	0.00271^a^	0.00148	27
**Market**			
Baseline	0.0030^a^		30
Cool	0.0029^a^	−0.0001	29
Cold	0.0033^a^	0.0003	33
Very cold	0.0043^a^	0.0013	43
**Cull sows**			
Baseline	0.0027^a^		27
Cool	0.0022^a^	−0.0005	22
Cold	0.0023^a^	−0.0004	23
Very cold	0.0029^a^	0.0002	29

*Estimated CR are based on a zero-inflated negative binomial generalized linear mixed model*.

*^a^Significant at alpha = 0.05. All heat index (HI) categories are nested within swine classes. For example, a significant proportion of variability in “dead condemnations” was accounted for by the BASELINE HI category nested within the ROASTER class after accounting for the offset term, log of total swine, and all other factors in the model*.

*^b^Minimum temperature categories of very cold, cold, and cool ranges are −17 to 9°F, 10–39°F, and 40–50°F, respectively. The baseline temperature category includes average temperatures of 54–79°F*.

Roaster and market swine classes were more negatively affected than cull sows by a decrease in minimum temperature compared to baseline. For every 10,000 roaster swine that go to slaughter in a week, our model suggests that we can expect to lose approximately 14, 19, and 27 of those swine due to “dead” condemnations at minimum temperature ranges that are cool (40–50°F), cold (10–39°F), and very cold (−17 to 9°F), respectively (Table 5). For every 10,000 market swine that go to slaughter in a week, we can expect to lose approximately 29, 33, and 43 of those swine due to “dead” condemnations at minimum temperature ranges that are cool, cold, and very cold, respectively. And for every 10,000 cull sows that go to slaughter, we can expect to lose approximately 22, 23, and 29 of those due to “dead” condemnations at minimum temperature ranges of cool, cold, and very cold, respectively. The AR for roaster swine was 0.00148 and for market 0.0013 when moving from our baseline category to our coldest temperature category. For these two swine classes, the AR generally increased as we moved to colder temperature categories. The cull sows actually had a reduced AR compared to baseline up until the coldest temperature range where it was 0.0002.

Setting our baseline to be the observations with average temperatures ranging from 54 to 79, the risk of condemnation (Table 6) is 1.21 times that of the roaster swine within the cool minimum weekly average. The cold and very cold minimum weekly averages have a risk of 1.55 and 2.20 times that of the baseline, respectively.

**Table 6:** **The risk ratio (RR) and 95% confidence interval range of “dead” condemnations by minimum temperature category in 149 slaughter plants with temperature data within 100 miles of plant location for United States Swine 2010-2015**.

Minimum temperature^a^ (°F)	RR	Confidence interval
**Roaster**		
Baseline	1.00	
Cool	1.21	(1.10, 1.33)
Cold	1.55	(1.41, 1.70)
Very cold	2.20	(1.98, 2.44)
**Market**		
Baseline	1.00	
Cool	0.97	(0.86, 1.09)
Cold	1.10	(0.98, 1.24)
Very cold	1.43	(1.26, 1.63)
**Cull sows**		
Baseline	1.00	
Cool	0.81	(0.72, 0.91)
Cold	0.85	(0.76, 0.95)
Very cold	1.07	(0.95, 1.21)

*^a^Minimum temperature categories of very cold, cold, and cool ranges are −17 to 9°F, 10–39°F, and 40–50°F, respectively. The baseline temperature category includes average temperatures of 54–79°F*.

For market swine with an average minimum temperature within the cool, cold, and very cold ranges, the risk of condemnation due to “dead” is 0.97, 1.10, and 1.43 times that of baseline, respectively. For cull breeding swine with a minimum temperature average within the cool, cold, and very cold categories, the risk of condemnation due to “dead” is 0.81, 0.85, and 1.07 times that of baseline, respectively.

Foregone revenue, or revenue not realized due to swine condemnations, in each of the temperature categories (Table 7) is more severely impacted by additional condemnations in hotter HI ranges on a per-pig basis, but cumulatively higher levels of foregone revenue are incurred in lower temperatures compared to the baseline due to higher numbers of swine condemned. Across the 2010–2015 study period, cumulative foregone revenue for all swine was $18.6 million and $4.3 million for cold temperatures and high HI ranges above the baseline, respectively. On average annually that is $3.1 million per year in foregone revenue for cold temperature ranges and $72,000 per year in foregone revenue for high HI ranges.

**Table 7:** **Increased condemnations in observed data above the expected value provided by a zero-inflated negative binomial generalized linear mixed model excluding hot and cold temperatures, the foregone revenue (in USD) from those condemnations, the total amount of swine in each swine and temperature category, and estimated percent of those swine that were condemned due to extreme temperatures in 149 slaughter plants with temperature data within 100 miles of plant location for United States Swine in 2010–2015**.

Temperature category^a^	Increased condemns	Foregone revenue (in USD thousands)	Swine in category (in thousands)	Increased swine condemned (%)
**Roaster**				
Very cold	260	$14.6	134	0.194
Cold	2,303	$124.1	1,236	0.186
Cool	1,256	$71.3	897	0.140
Total	3,820	$210.0	2,267	0.169
**Roaster**				
Moderate	125	$7.9	136	0.092
Hot	62	$4.4	161	0.039
Very hot	1	$0.05	51	0.001
Total	188	$12.2	348	0.054
**Market**				
Very cold	11,650	$1,945.1	46,109	0.025
Cold	46,913	$8,231.3	216,300	0.022
Cool	22,790	$4,210.6	123,649	0.018
Total	81,353	$14,388.1	386,058	0.021
**Market**				
Moderate	5,917	$1,165.4	8,350	0.071
Hot	10,720	$2,075.9	14,432	0.074
Very hot	283	$53.7	2,102	0.014
Total	16,920	$3,295.0	24,884	0.068
**Cull sows**				
Very cold	834	$231.1	520	0.160
Cold	7,131	$1,793.6	6,057	0.118
Cool	7,500	$1,982.3	4,133	0.181
Total	15,515	$4,007.0	10,710	0.145
**Cull sows**				
Moderate	992	$262.7	368	0.334
Hot	2,275	$538.2	682	0.269
Very hot	814	$211.6	154	0.528
Total	4,081	$1,012.4	1,204	0.229

*^a^Heat index categories of baseline, moderate, hot, and very hot ranges are 50–79°F, 80–84°F, 85–92°F, and 93–106°F, respectively. Minimum temperature categories of very cold, cold, and cool ranges are −17 to 9°F, 10–39°F, 40–50°F, respectively. The baseline temperature category includes average temperatures of 54–79°F*.

**Figure 4:**
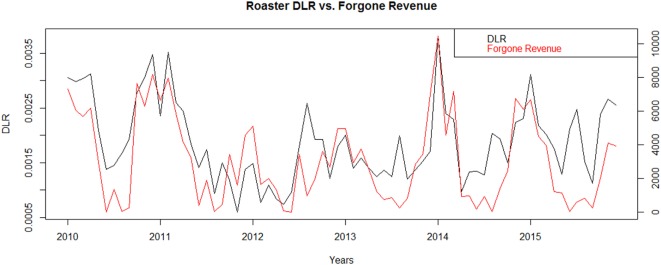
“Dead” loss ratio (DLR)^†^ compared to Forgone Revenue* of Roaster swine in 69 slaughter plants with temperature data within 100 miles of plant location for United States from 2010 to 2015. *Forgone Revenue is estimated from increased condemnations in observed data above the expected value provided by a zero-inflated negative binomial generalized linear mixed model excluding hot and cold temperatures. ^†^DLR calculated by number of “dead” condemnations divided by total swine multiplied by 100.

Roaster condemnations result in the lowest levels of foregone revenue per head due to their small carcass size. Across the 2010–2015 period, cumulative foregone revenue was $210,000 and $12,000 in cold and hot temperature categories above the baseline, respectively. Looking across years in Figure 4, roaster swine foregone revenue follows similar patterns seasonally as the DLR where foregone revenue due to condemnations peaks in the winter months.

**Figure 5:**
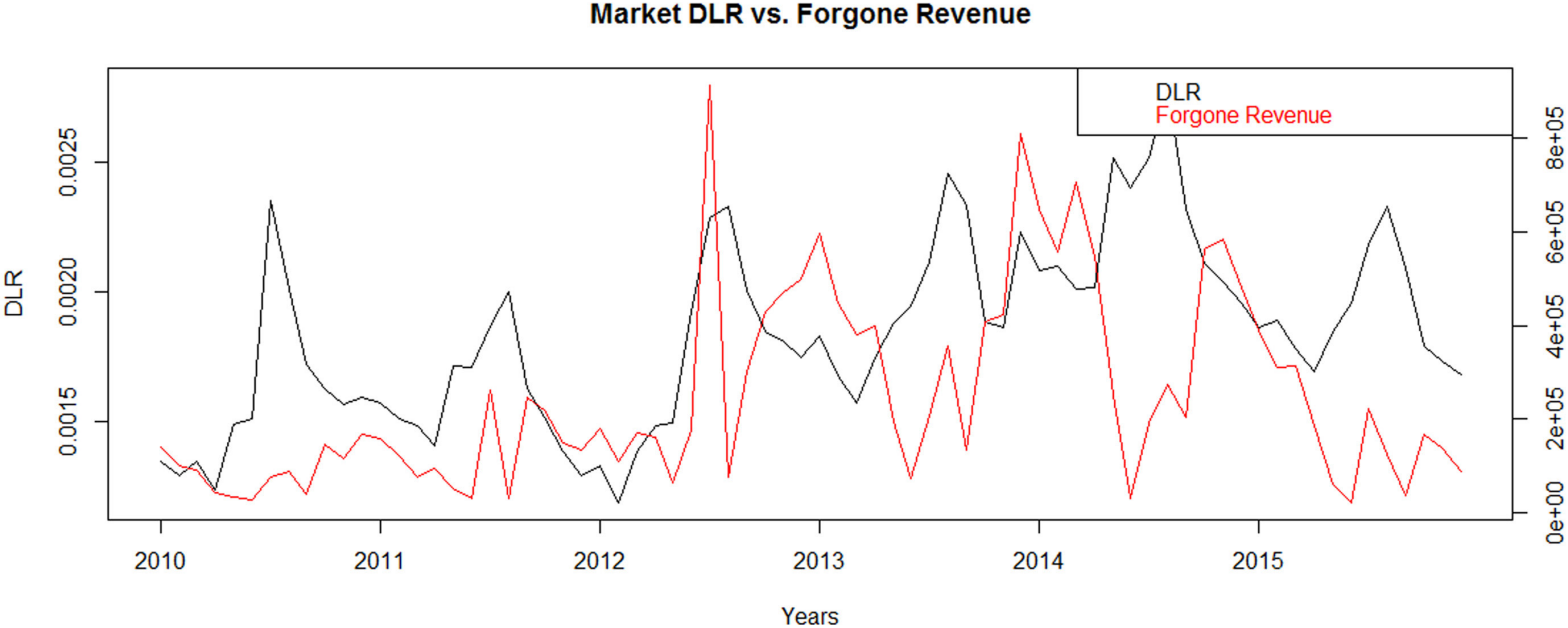
“Dead” loss ratio (DLR)^†^ compared to Forgone Revenue* of Market swine in 123 slaughter plants with temperature data within 100 miles of plant location for United States from 2010-2015. *Forgone Revenue is estimated from increased condemnations in observed data above the expected value provided by a zero-inflated negative binomial generalized linear mixed model excluding hot and cold temperatures. ^†^DLR calculated by number of “dead” condemnations divided by total swine multiplied by 100.

Of the three swine categories in the study period, market swine resulted in the highest levels of foregone revenue, due to their relatively high value and large finished weights. Total foregone revenue of $14.4 million and $3.3 million were accumulated for cold temperature ranges above the baseline and hot temperature ranges above the baseline, respectively. This means that on average each year, the increases in condemnations due to cold temperatures would be expected to result in a loss of almost $2.5 million, and condemnations due to an elevated HI would be expected to result in a loss of about half a million dollars. Looking across years in Figure 5, the foregone revenue for market swine often diverges from the pattern in the DLR. This likely reflects the impact of seasonal price patterns and exogenous market drivers. Typically pork prices peak in the summer, but the greatest consumption of pork, and the greatest amount of slaughter, occurs at the winter holidays in NA and Easter.

**Figure 6:**
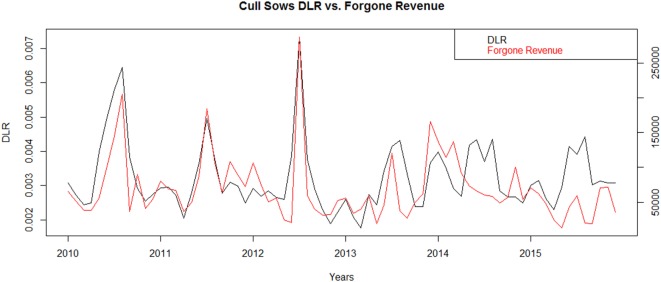
“Dead” loss ratio (DLR)^†^ compared to Forgone Revenue* of Cull Sow swine in 46 slaughter plants with temperature data within 100 miles of plant location for United States from 2010 to 2015. *Forgone Revenue is estimated from increased condemnations in observed data above the expected value provided by a zero-inflated negative binomial generalized linear mixed model excluding hot and cold temperatures. ^†^DLR calculated by number of “dead” condemnations divided by total swine multiplied by 100.

Condemnations of cull sows resulted in accumulated foregone revenue of $4 million and $1 million for cold temperature ranges above the baseline and hot temperature ranges above the baseline, respectively, across the 6-year study period. On average each year, the increases in condemnations due to cold temperatures would be expected to result in a loss of almost $668,000, and condemnations due to hot temperatures would be expected to result in a loss of $169,000. Like roaster pigs, foregone revenue across years (Figure 6) mostly follows the pattern of the DLR, peaking in winter months.

Seasonal variation in market prices results in a more complex impact on cumulative foregone revenue for market swine and cull sows across the 6 years. For market swine with an average HI within moderate or hot ranges, the foregone revenue due to “dead” condemnations is $1.2 million and $2.1 million, respectively. For the very hot HI range, the weekly average foregone revenue is only $54,000. For market swine with an average minimum temperature within cool, cold, and very cold ranges the foregone revenue due to “dead” condemnations is $4.2 million, $8.2 million, and $1.9 million, respectively. Price per head for market swine peaks annually in the months with higher temperature ranges, meaning foregone revenue can be higher in the heat extreme temperature ranges even though the increase in condemns is lower compared to the cold extreme temperature ranges.

For cull sows with an average HI within moderate, hot, or very hot ranges, the cumulative foregone revenue due to “dead” condemnations across the study period is $263,000, $538,000, and $212,000, respectively. For cull sows with an average minimum temperature within cool, cold, and very cold ranges, the foregone revenue due to “dead” condemnations is $2 million, $1.8 million, and $231,000 respectively. Cull sows experience a different seasonal price variation pattern than market hogs.

## Discussion

There are many factors that influence mortality during transport to slaughter. Two significant factors are the ambient temperature during the journey and the genetic make-up of the pigs related to their response to stress and ability to cope with high temperatures (15, 16). Other factors include whether the pigs are fasted before the journey, distance/duration of the journey, loading/unloading procedures and time, truck type and pig density. Most of the literature on heat/cold stress and transport loss addresses effects in market swine with very little concerning sow or roaster swine classes.

Averos et al. (15) found not fasting market pigs doubled the risk of mortality, and the duration of the journey had little effect when the pigs had been fasted. An increase in market swine mortality with increasing transport distance was reported by both Voslarova et al. (3) and Haley et al. (4). Sutherland et al. (2) reported the percentage of dead market pigs increased as the journey time increased from 30 min to 4 h, but decreased during journeys lasting more than 4 h.

Stress during loading varies with the farm of origin due to different loading procedures, truck type, chute arrangements and prod types used. Haley et al. (4) and Fitzgerald et al. (11) found a consistent influence of farm management style on in-transit market pig losses. In a study by Averos et al. (15) conducted in 5 EU countries the use of electric prods and the presence or absence of bedding were not significant risk factors for mortality, but an increase in the average loading time per pig reduced the risk of mortality during the journey. Sutherland et al. (2) found that the presence and condition of bedding (wet or dry) affected the percentages of dead market pigs during transport.

Correa et al. (17) and Conte et al. (18) found that the truck type and animal location in the truck affect the welfare of market pigs during transport. Potbelly trailers have three decks and steep internal ramps compared to straight double deck trucks, and have been associated with a higher proportion of dead and fatigued pigs compared to other truck types (7, 19). The negative impact on animal welfare of some locations in a potbelly trailer were more pronounced in the winter due to the additive effect of cold stress (17). In contrast, Ritter et al. (20) and Kephart et al. (21) found that there was no significant effect of trailer type (potbelly versus straight deck) on market pig death losses during transport, although the potbelly trailer was associated with more open-mouth breathing and skin discoloration at unloading.

The loading density on the truck affects animal welfare and meat quality; however, in NA, there is lack of consensus on loading density recommendations and how much loading densities should be reduced at higher temperatures (7). In a study of factors associated with fatigued, injured, and dead market pig frequency during transport and lairage at a commercial slaughter facility in the Midwestern US, trailer density accounted for the largest portion of variation in total losses per trailer load (11). Haley et al. (5) found at temperatures <70°F in-transit losses of market pigs increased 2.12 times when space allowances were between 0.44 and 0.43 m^2^/pig compared with ≥0.515 m^2^/pig, but concluded that temperature is likely a more important determinant of in-transit loss than space allowance.

This retrospective observational study used ambient temperature and humidity to predict the incidence and risk of death among swine in-transit and in lairage pens just prior to slaughter. Potentially important variables that might modify the relationship between “dead” condemnations and temperature such as swine class (market, roaster, and cull sows) and slaughter establishment processing volume were included in the analysis. Foregone revenue was estimated for swine classes at different temperature extremes using weekly swine prices.

Our incidence of death in-transit and during lairage for all swine classes combined from 2010 to 2015 of 0.19% is in the range reported by others for NA. Of all pigs marketed in the US from 2000 to 2006, 0.22% died during transport to slaughter in the US (6). From June to August 2003 in Eastern Canada, the incidence of market pig deaths in-transit was 0.22% (5). The incidence of deaths in-transit in some EU countries is reported to be somewhat less than in NA. Voslarova et al. (3) found in the Czech Republic from 2009 to 2014 in-transit deaths of market pigs varied by distance traveled, ranging from 0.049% in pigs transported for distances below 50 km to 0.145% for distances over 300 km. Averos et al. (15) reported an average incidence of market pig death in-transit of 0.11% between June 2003 and May 2004 in 5 EU countries.

Observing our RR for increasing HI, as expected, we found large cull breeding swine experienced a significant increase in the risk of “dead” condemnations when transported during increasingly hot HI categories (Table 4). Unexpectedly, roaster swine showed a different pattern altogether, with decreasing risk at higher HI values. Some possible explanations for this unexpected finding are the (a) smaller size of roaster pigs may make them more capable of dissipating heat and coping with a higher HI and (b) they may be stocked less densely on transport trailers, giving them more space and less pig-to-pig contact, allowing them to stay cooler (4). Mitigation measures already in place may be more effective for roaster and market swine than larger swine classes such as sows. Cull sows are older and may travel farther to slaughter (due to fewer plants that make sow meat products) than other swine classes and therefore have a greater risk for welfare problems (22). In addition, genetic selection of sows that has allowed significant improvements in reproductive traits has also increased metabolic heat production resulting in a decrease in the sow’s ability to cope with high ambient temperatures (23, 24).

Observing our RR for decreasing minimum temperature, we found varying results (Table 6). The smaller roaster swine were the only ones to show a consistent trend, where their risk of condemnation increased as the minimum temperature decreased. Market swine seemed to tolerate the cold fairly well, they did not experience a significant change in risk compared to baseline until the coldest temperature category. In cull sows we saw a significant decrease in risk for the cool and cold categories compared to baseline, but in the coldest category the risk was similar to baseline. The result for the market and cull sows may be due to the range of the baseline temperature including temperatures that can still be considered hot to some, but not enough to make it to the minimum HI cutoff. Due to this, some of the condemnations from market and cull sows in the baseline may have been due to warmer temperatures.

Cumulative foregone revenue from condemnations was lowest for the smallest, lower value roaster swine (Table 7). Foregone revenue from increases in market swine condemnations are not necessarily intuitive. The highest probability of increases in swine condemnations as a percent of swine slaughtered occurs in the hottest temperature ranges. The total volume of slaughter is higher in the coldest temperature ranges. Thus, the cumulative foregone revenue is highest for the cold temperature ranges. However, the marginal foregone revenue—equal to the price per head of market swine in this study—is higher in the hot temperature categories reflecting the seasonal peaks in hog prices. For example, in Table 7, the coldest temperature category results in a greater increase in market swine condemnations compared to the baseline than the hot temperature category compared to the baseline. However, due to seasonal price differences driven by product demand, the foregone revenue for the additional market swine lost due to hot HI values is greater compared to the baseline. Thus the decision maker is left to consider whether the goal in adopting extreme temperature stress mitigation measures is to reduce the overall foregone revenue or the marginal loss for each additional hog condemned. If the former, cold temperature stress mitigation strategies will be targeted. If the latter, heat stress mitigation strategies will be targeted.

Cull sows vary only slightly from market swine because these swine are often used for different pork products that have different seasonal demands. But trends in numbers of head slaughtered are driven by expansion and contraction in the sow herd inventory and are similar to market swine slaughter trends. Like market swine, the slaughter volumes result in the highest cumulative foregone revenue in cold weather ranges, but market price patterns can result in higher levels of marginal foregone revenue in warmer temperature ranges due to seasonal peaks in prices driven by product demand. This further emphasizes the need to mitigate heat stress for those swine categories most susceptible.

A previous study (6) found that deaths in transport (from any cause) in 2006 were $24 million across all swine classes. These losses were based on net income loss rather than gross income loss reported here. Our study indicates that some of these annual revenue losses may be avoidable through mitigation measures for cold and heat stress in swine in transport. Further, the levels of foregone revenue suggest that investment in mitigation measures costing less annually than the annual foregone revenue from temperature-related condemnations should net additional income for swine producers and processors. Some mitigation measures such as bedding, fans, water sprinkling, and decreasing pig density are not that costly. Some larger investments such as refrigerated trucks may be cost prohibitive. Effective ventilation and water sprinkling in a stationary truck have been shown to reduce deaths during transportation (7). Nannoni et al. (25) found that a water sprinkling system inside a potbellied truck and activated for 5 min in the stationary truck both at the farm when loading was complete and again at the slaughter plant before unloading, was effective in reducing the stress response and improving the pork quality of market pigs transported in potbellied trailers, particularly in some of the trailer compartments. Fox et al. (26) reported that at ambient temperatures >23°C the gastrointestinal temperature of water sprinkled market pigs compared to non-sprinkled pigs was lower, they drank less often in lairage and there were no detrimental effects on unloading procedures.

Limitations of this study include the inability to account for other factors related to death during transit such as loading conditions, length and duration of the journey, the density of pigs on the truck, genetics, and the use of mitigation measures such as ventilation, sprinklers, fans, and bedding. Because we only had weekly condemnation data available, we could not assess the range of variability in temperature during the week and we can not make conclusions about the impact of individual days with high HI or low minimum temperature values. Further, the economic measure used here, foregone revenue, is limited since it does not reflect the potential changes in prices resulting from fewer swine expiring in transit or while waiting for slaughter. Future studies should include a more expansive market analysis from reduced temperature-related condemnations.

This paper adds to the current body of knowledge by providing information on the effects of extreme temperatures on “dead” condemnations across swine classes. In addition, estimates of foregone revenue due to temperature extremes were lacking in the literature. As a result of this study, weekly estimated HI values and ambient temperature data for slaughter establishments can be incorporated into the USDA-APHIS swine condemnation surveillance to provide additional information for analysts investigating signals (noteworthy increases above baseline) for “dead” condemnations by swine class. This study suggests that current mitigation measures are often not sufficient to prevent swine deaths due to ambient temperature extremes. These deaths are an animal welfare concern and also represent lost revenue for the swine industry (6). Foregone revenue results indicate that incentives may exist to invest in extreme temperature stress mitigation strategies to reduce temperature-related deaths by examining, for the first time, amounts of revenue not achieved specifically due to heat and cold stress. Further, incentives to invest in mitigating heat stress or cold stress are different depending on whether the goal is to reduce cumulative foregone revenue due to temperature-related deaths or the marginal foregone revenue for each additional hog that dies due to extreme temperatures in transit. If the former, the incentive is to invest in cold stress mitigation when greater volumes of hogs are going to slaughter. If the latter, the incentive is to invest in heat stress mitigation when the price per hog is greater. Improvements in the use and effectiveness of mitigation measures should be investigated.

## Authors Contributions

JA conceived the study. EP analyzed the data. JA, EP, MR, and AH participated in study design, coordination, and interpretation of output. EP, JA, and AH drafted the manuscript. All authors read and approved the final manuscript.

## Conflict of Interest Statement

The authors declare that the research was conducted in the absence of any commercial or financial relationships that could be construed as a potential conflict of interest.

## Footnotes

^1^https://www.weather.gov/media/ffc/ta_htindx.PDF.

^2^www.lmic.info.
